# Characterization of a putative grapevine Zn transporter, VvZIP3, suggests its involvement in early reproductive development in Vitis vinifera L

**DOI:** 10.1186/1471-2229-12-111

**Published:** 2012-07-23

**Authors:** Felipe Gainza-Cortés, Ricardo Pérez-Dïaz, Ramón Pérez-Castro, Jaime Tapia, José A Casaretto, Sebastián González, Hugo Peña-Cortés, Simón Ruiz-Lara, Enrique González

**Affiliations:** 1 Instituto de Biología Vegetal y Biotecnología, Universidad de Talca, Talca, Chile; 2 Instituto de Química de Recursos Naturales, Universidad de Talca, Talca, Chile; 3 Centro de Biotecnología Daniel Alkalay Lowitt, Universidad Federico Santa María, Valparaíso, Chile; 4 Centro de Estudios Avanzados en Fruticultura (CEAF) CONICYT-Regional, GORE-O’Higgins R08I1001, Rengo, Chile; 5 Laboratorio de Investigaciones Biomédicas, Facultad de Medicina, Universidad Católica del Maule, Talca, Chile

## Abstract

**Background:**

Zinc (Zn) deficiency is one of the most widespread mineral nutritional problems that affect normal development in plants. Because Zn cannot passively diffuse across cell membranes, it must be transported into intracellular compartments for all biological processes where Zn is required. Several members of the Zinc-regulated transporters, Iron-regulated transporter-like Protein (ZIP) gene family have been characterized in plants, and have shown to be involved in metal uptake and transport. This study describes the first putative Zn transporter in grapevine. Unravelling its function may explain an important symptom of Zn deficiency in grapevines, which is the production of clusters with fewer and usually smaller berries than normal.

**Results:**

We identified and characterized a putative Zn transporter from berries of *Vitis vinifera L.*, named VvZIP3. Compared to other members of the ZIP family identified in the *Vitis vinifera L.* genome, *VvZIP3* is mainly expressed in reproductive tissue - specifically in developing flowers - which correlates with the high Zn accumulation in these organs. Contrary to this, the low expression of *VvZIP3* in parthenocarpic berries shows a relationship with the lower Zn accumulation in this tissue than in normal seeded berries where its expression is induced by Zn. The predicted protein sequence indicates strong similarity with several members of the ZIP family from Arabidopsis thaliana and other species. Moreover, VvZIP3 complemented the growth defect of a yeast Zn-uptake mutant, ZHY3, and is localized in the plasma membrane of plant cells, suggesting that VvZIP3 has the function of a Zn uptake transporter.

**Conclusions:**

Our results suggest that VvZIP3 encodes a putative plasma membrane Zn transporter protein member of the ZIP gene family that might play a role in Zn uptake and distribution during the early reproductive development in *Vitis vinifera L.*, indicating that the availability of this micronutrient may be relevant for reproductive development.

## Background

Zinc is an essential micronutrient that plays many important roles in various physiological and metabolic processes in all living organisms. It functions as a cofactor for over 300 enzymes and proteins involved in cell division, nucleic acid metabolism and protein synthesis, and is critical in the control of gene transcription and the coordination of other biological processes regulated by proteins containing DNA-binding Zn-finger motifs, RING fingers and LIM domains [1-4].

It has been demonstrated that Zn deficiency is one of the most widespread mineral nutritional problems affecting normal development in plants [5-8]. This includes altered expression and/or function of proteins at the metabolic level that leads to different physiological symptoms characterized by root apex necrosis. At the same time, sub-lethal Zn deficiency induces spatial heterogeneous or interveinal chlorosis, development of reddish-brown or bronze tints and a range of auxin deficiency-like responses such as internode shortening, epinasty, inward curling of leaf lamina and reduction of leaf size [4-9]. In grapevines, deficit of Zn results in the development of leaves that are smaller than normal and/or mottled, and shortened internodes. It has been suggested that this reduction in shoot growth results from the fact that Zn is essential for the synthesis of tryptophan, a precursor of the phytohormone indoleacetic acid (IAA) [10]. Another important symptom of Zn deficiency in grapevines is the production of clusters with few berries that also vary in size from normal to very small [10-12]. In this way, vineyards commonly correct Zn deficiency with both soil and foliar application of fertilizers. Under conditions of Zn deficiency, application of foliar Zn fertilizer shortly before anthesis increases the number of flowers that set fruit [10-14].

Since Zn cannot passively diffuse across cell membranes, it must be transported into intracellular compartments for all biological processes where Zn is required. Several members of the 15 Zinc-regulated transporters, Iron-regulated transporter-like Protein (ZIP) gene family have been characterized in *Arabidopsis thaliana*[15], and their involvement in metal uptake and transport in plants has been demonstrated [16-18].

Arabidopsis *ZIP1* and *ZIP3* genes are expressed in roots in response to Zn deficiency, suggesting that they transport Zn from the soil to the plant. *ZIP4* is expressed either in roots and shoots, showing a delicate regulation to control the homeostasis of Zn, thus avoiding potential toxic effects of this micronutrient [15,17]. Additionally, these three transporters restore Zn uptake in the yeast Zn-uptake mutant, *Δzrt1*/*Δzrt2* (ZHY3 strain), confirming its implication in Zn homeostasis [15,19-22]. Moreover, *ZIP2* and *ZIP4* can rescue yeast mutants deficient in copper (Cu) transport, and *ZIP4* is up-regulated in Cu-deficient roots [23]. Although several ZIP genes have been identified and functionally characterized at the molecular level [2,24], the complete gene family and their role in metal homeostasis is not fully understood. In this way, a considerably large family of ZIP genes has been currently characterized from various species, such as *Thlaspi japonicum*[25], *Thlaspi caerulescens*[17,26-28], soybean [29], *Medicago truncatula*[30,31] and rice [2,24,32,33]. The availability of the full-genome sequence of grapevine (http://www.genoscope.cns.fr/spip/Vitis-vinifera-e.html) provides the opportunity to investigate the Zn homeostasis and their importance for the reproductive development in this organism. 20 ZIP genes have been identified recently in *Vitis vinifera*[34], but none of them has been functionally characterized.

Here, we report the isolation of *VvZIP3*, a member of the ZIP gene family from *Vitis vinifera L.* cv. Carménère, which encodes a Zn uptake protein. This gene was isolated from an expression library of Carménère berries and is highly homologous to *AtZIP1* gene *from Arabidopsis thaliana*. Expression of *VvZIP3* in the yeast Zn-uptake deficient mutant ZHY3 complemented its growth defect, indicating that VvZIP3 has the function of a putative Zn transporter. Moreover, the expression of the VvZIP3-mGFP5 fusion protein in onion epidermal cells indicated that is located at the plasma membrane. Expression analysis revealed that *VvZIP3* was transcribed principally in reproductive tissues - specifically in developing flowers -, organs that also present the highest accumulation of Zn. These results suggest that VvZIP3 participates in Zn uptake during flower development contributing to the normal development in *Vitis vinifera L*.

## Results

### *VvZIP3 is a member of a Zn transporter encoding gene family*

**Figure 1:**
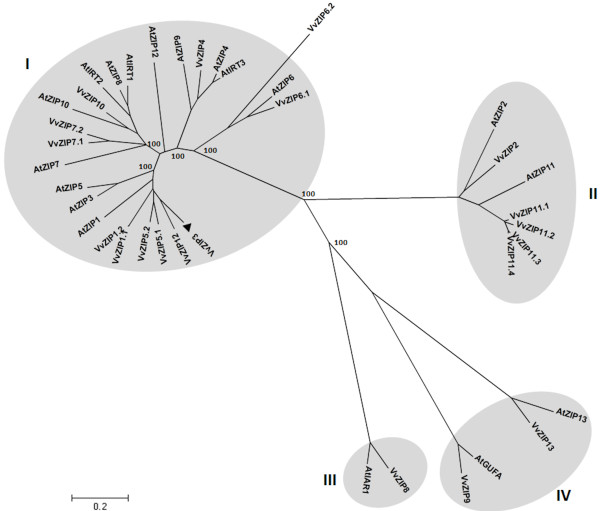
**Phylogenetic relationship between VvZIP3 and ZIP proteins from Vitis and Arabidopsis thaliana.** Phylogenetic analysis was performed using the software MEGA 4.0 (http://www.megasoftware.net/mega.html). It is assumed that the length of the branches is proportional to phylogenetic distances. Position of VvZIP3 in the not rooted tree is marked by a black arrowhead. Grapevine protein sequences were deduced from the nucleotide sequence obtained in this work (VvZIP3) or from the GenBank accessions (see methods).

In a previous preliminary study, a macroarray containing approximately 4800 ESTs from grapevine reproductive tissue expression libraries was screened to compare the transcriptomic profiles of normal and seedless berries from Carménère cultivar [35]. A gene coding for a protein similar to a metal transporter from *Medicago truncatula* was found to be strongly repressed in seedless berries (see Additional file 1). The corresponding full length cDNA was isolated from an expression library of Carménère berries and its sequence was subjected to different *in silico* analyses for further characterization. When compared with the ZIP genes identified in the grapevine genome [34] the isolated gene was found to correspond to *VvZIP3.* Sequence analysis and comparison of the cDNA with genomic DNA shows that *VvZIP3* is composed of three exons and two introns, being a single copy gene located in chromosome I of grapevine (data not shown). A phylogenetic tree was obtained compiling VvZIP3 protein with other sequences of known *Arabidopsis thaliana* and the recently identified *Vitis vinifera L.* ZIP members (Figure 1). This association revealed that VvZIP3 is closely related to AtZIP5, AtZIP3 and AtZIP1, three characterized Zn transporters in *Arabidopsis*[15].

### *VvZIP3 is differentially expressed during grapevine development*

**Figure 2:**
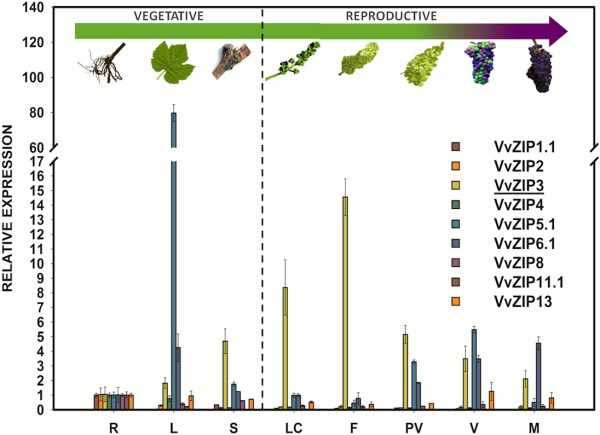
**Gene expression analysis of members of*Vitis vinifera L. ZIP*gene family.** Expression profiles of representative members of Vitis vinifera ZIP gene family in R (roots); L (leafs); S (stems); LC (little clusters); F (flowers); PV (pre-veraison fruit); V (whole veraison fruit); M (whole mature fruit). Expression in root samples was adjusted to 1 relative unit. The end of vegetative development and the beginning of reproductive development is divided by a dotted line. The images are representations of each phenological stage. VvZIP3 is underlined. Data represent means ± SD (n = 3).

As a first approximation to determine the expression profile of *VvZIP3* and its relevance during the development of *Vitis vinifera L.* cv. Carménère, relative expression level was measured by qPCR and compared with other representative ZIP genes identified in the grapevine genome [34]. Based on clusters determined in the phylogenetic comparison (Figure 1), *VvZIP1.1VvZIP2VvZIP4VvZIP5.1VvZIP6.1VvZIP8VvZIP11.1* and *VvZIP13* were selected for this analysis. Total RNA was isolated from roots, leaves, stems, little clusters, flowers, fruits and seeds at different developmental stages (from pre-veraison to mature grapes) during the S3 growing period, covering important events such as flower and berry development. This analysis reveal that *VvZIP5.1* is mainly expressed in leaves during the vegetative development (about 80-fold compared to roots) but its expression decay during early stages of the reproductive development, being up-regulated again at pre-veraison (PV) and veraison (V) stages but to a lesser extent than in leaves (Figure 2). On the other hand, *VvZIP3* presented a differential expression profile in both vegetative and reproductive tissues, characterized for a high expression during reproductive development. While in vegetative tissues the expression prevailed mainly in stems and at low levels in roots and leaves, in reproductive tissues *VvZIP3* was mainly expressed in little cluster and flowers, with a significant up regulation (about fifteen-fold) in flowers. Contrary to this, reduction of the *VvZIP3* transcripts was evident in berries as maturation stages progressed (Figure 2), suggesting that *VvZIP3* could be important during the early stages of the reproductive development in *Vitis vinifera L.* Other members of *VvZIP* family analyzed shown a low expression level except for *VvZIP6.1* that is induced as berry maturation stages progressed (Figure 2).

### *VvZIP3 protein has conserved motifs associated to Zn transporters*

**Figure 3:**
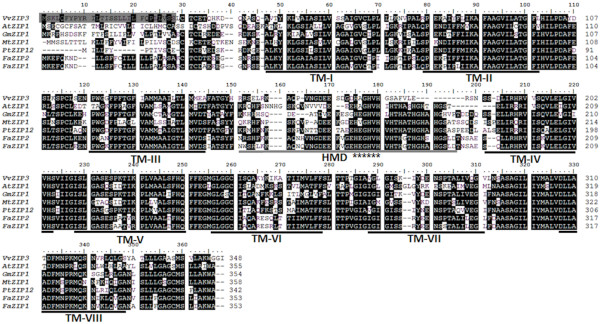
**Comparison of the VvZIP3 amino acid sequence with its homologues from other plant species.** Alignment was performed with highest similar homologous sequences. Identical residues are blackened. The eight transmembrane domains are highlighted by black lines under the sequences and numbered from I to VIII, as predicted by TMHMM and TMpred (see methods). HMD, highlighted by light gray line, depicts the histidine-rich metal-binding domain. A potential metal binding motif highly conserved (HxGHVH) is marked between TM-III and TM-IV with asterisks under the sequences. The N-terminus line under the VvZIP3 sequence (grey box) highlights a possible signal peptide for plasma membrane localization obtained by WOLF-PSORT-II software (see methods).

**Figure 4:**
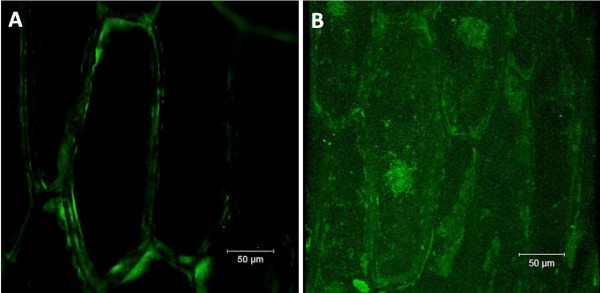
**Cellular localization of VvZIP3-mGFP5 in planta.** Transient expression of **(A)** VvZIP3-mGFP5 and **(B)** mGFP5 constructs in onion epidermal cells. Both genetic constructs were incorporated by particle bombardment and transformed bulb scale epidermal layers were incubated for 16 h at 25°C before visualization by confocal microscopy (Bars = 50 μm).

BLAST search on the translated protein sequence indicated strong homology with several members of the ZIP family from *Arabidopsis thaliana* as well as with other Zn and iron (Fe) transporters (Figures 1 and 3). Multiple alignments of the translated sequence with homologous proteins showed that *VvZIP3* encodes a protein of 348 amino acid residues (of ca. 37.3 KDa). In agreement with the structure of other ZIP protein family members, principally AtZIP1 (accession AT3G12750), ZIP1 and ZIP2 from *Fragaria X ananasa* (AAX28838 and AAX28837, respectively), GmZIP1 (AAK37761), PtZIP12 (XP_002315075) and MtZIP1 (AAR08412), VvZIP3 was predicted to contain eight transmembrane (TM) domains, a very short C-terminal tail, and a hydrophilic region between TM domains III and IV, being this the most variable region in length and containing a potential metal-binding domain rich in histidine residues (Figure 3). This region was predicted to be directed toward the inside surface of the membrane. Further analyses with the Wolf PSORT-II software [36] (http://wolfpsort.org/) showed that VvZIP3 is predicted to be a plasma membrane protein with a potential signal peptide in the first 28 residues (Figure 3). The cellular localization assigned by *in silico* analysis was experimentally tested. *VvZIP3* cDNA fused to the N-terminal coding part of the modified green fluorescent protein 5 (mGFP5) was transiently expressed under the control of the cauliflower mosaic virus (CaMV) 35 S promoter in onion epidermal cells. The fluorescence of the *VvZIP3*-mGFP5 fusion protein was observed at the plasma membrane (Figure 4A), while that of mGFP5 alone was localized to the cytoplasm and nucleus (Figure 4B), suggesting that *VvZIP3* is a transporter protein located at the plasma membrane.

### *VvZIP3 restores Zn-limited growth in the yeast ZHY3 double mutant strain*

**Figure 5:**
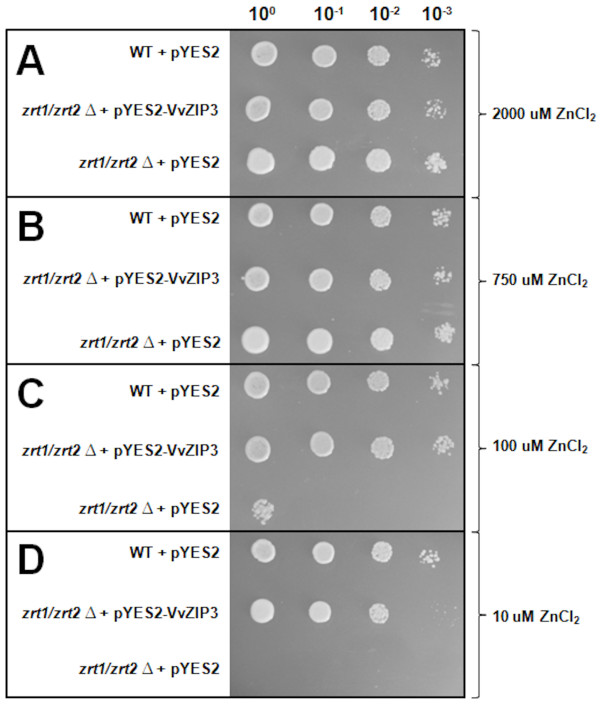
**Complementation of the ZHY3 (zrt1zrt2Δ) yeast mutant with VvZIP3.** The double mutant strain was transformed with either the empty vector pYES2 or with the pYES2-VvZIP3 construction. The wild type parental strain DY1457 (WT) was also transformed with pYES2 as a control. The transformed cells were adjusted to OD_600_ of 1 and 5 μl of serial dilutions (from left to right in each panel) and were spotted on SC-U plates supplemented with different concentrations of ZnCl_2_. Plates were incubated for 6 days at 30°C.

To support the role of VvZIP3 as a Zn uptake transporter, the yeast mutant ZHY3 [19,20] defective in Zn uptake, due to the inactivation of both its high (*Δzrt1)*- and low (*Δzrt2)*-affinity Zn transporters, was used in a complementation experiment. ZHY3 cells transformed with the empty pYES2 expression vector were able to grow on synthetically defined medium only when supplemented with Zn (750 μM to 2 mM) (Figure 5). However, ZHY3 transformed with the pYES2 expression vector containing the *VvZIP3* cDNA (pYES2-*VvZIP3*) grew well on media supplemented with both low (10 μM and 100 μM) and high Zn (750 μM and 2 mM). The wild type parental yeast strain DY1457 also developed on media with both low and high Zn concentrations (Figure 5). These results indicate that *VvZIP3* complemented the mutations of ZHY3, apparently by transporting Zn across the yeast plasma membrane.

### *VvZIP3 is mainly expressed in the pericarp and skin of berries and is related to normal fruit development*

**Figure 6:**
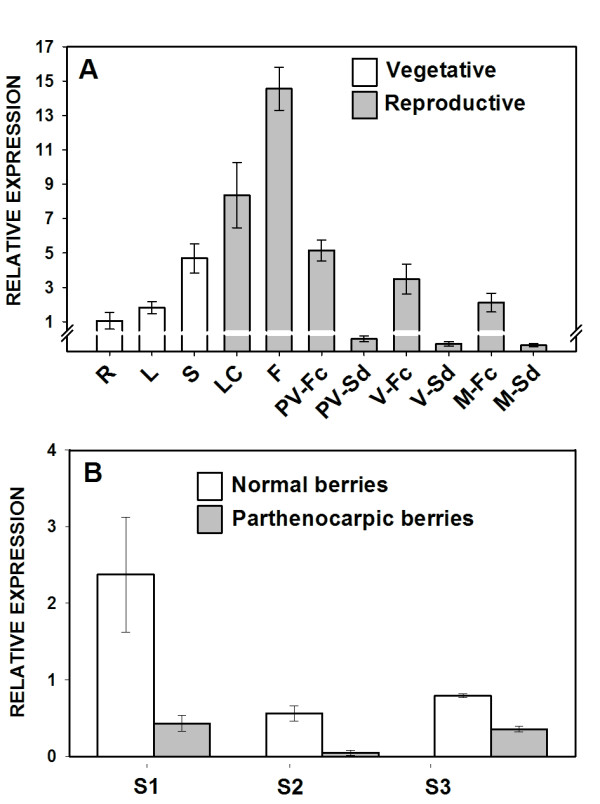
**Gene expression analysis of VvZIP3 in grapevine tissues. (A)** Expression analysis of *VvZIP3* in R (roots); L (leafs); S (stems); LC (little clusters); F (flowers); PV-Fc (pre-veraison fruit complete); PV-Sd (pre-veraison seeds); V-Fc (whole veraison fruit); V-Sd (veraison seeds); M-Fc (whole mature fruit); M-Sd (mature seeds). Expression in root samples was adjusted to 1 relative unit. Relative expression values of PV-Sd, V-Sd and M-Sd are 0.039, 0.024 and 0.019, respectively. Data represent means ± SD (n = 3). **(B)** Expression analysis of *VvZIP3* in normal and parthenocarpic berries at the pre-veraison stage during three growing seasons (S1, S2 and S3). qPCR analyses of *VvZIP3* expression was normalized against the expression level of *VvGAPDH*. Data represent means ± SD (n = 3).

A more detailed analysis of the transcriptional profile of *VvZIP3* along fruit development showed that the expression level during berry growth was significant lower than that in early stages of reproductive development (inflorescences and flowers) and appears to be restricted to the pericarp and skin tissues since a very low transcriptional activity was detected in seeds (Figure 6A).

In field conditions, Zn deficiency is commonly associated to the production of clusters with few berries that vary in size from normal seeded to very small or parthenocarpic unseeded berries, a viticulture problem known as *millerandage*[10-12]. In order to test whether *VvZIP3* expression is being affected in such phenotype, its transcriptional profile was compared in normal and parthenocarpic grapes in an early developmental stage (pre-veraison) from three different seasons (S1, S2 and S3). This analysis revealed that expression of *VvZIP3* was consistently repressed in parthenocarpic green berries compared to normal berries in the three growing seasons analyzed (Figure 6B). Taken together, these results suggest that *VvZIP3* is mainly expressed when the plant needs high requirement of Zn (flowering and fruit setting) and that its expression is associated with the physiological processes that affect the normal berry development in grapevine.

### *Zn accumulation in reproductive tissue of Vitis vinifera L. cv. Carménère is associated to VvZIP3 expression profiles*

**Figure 7:**
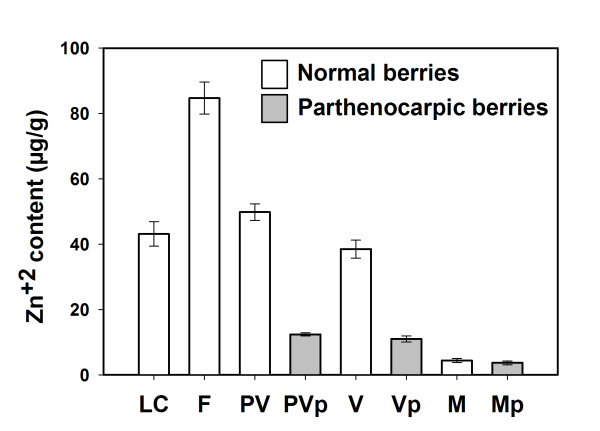
**Zn accumulation patterns in grapevine.** Zn concentration in developing organs of *Vitis vinifera L.* in season 3 (S3). LC (little clusters); F (flowers); PV (pre-veraison berries); PVp (pre-veraison parthenocarpic berries); V (veraison berries); Vp (veraison parthenocarpic berries); M (mature berries); Mp (mature parthenocarpic berries). Data represent means ± SD (n = 3).

To examine whether the Zn content profiles correlate with the expression of *VvZIP3* on the same season and to determine the profiles of accumulation of this metal during reproductive development, the concentration of Zn was measured in little clusters, flowers and fruits from season S3 (Figure 7). As expected, there was a significant increase of Zn levels in flowers, about two-fold compared to those in little clusters. This observation suggests that VvZIP3 plays a relevant role in Zn transport during flower development. After flowering stage, the reduction in Zn content was evident as maturation stages progressed. Additionally, parthenocarpic berries showed reduced Zn content when compared with normal berries before the maturation stage. These results seems to be consistent with the *VvZIP3* expression profile (Figure 6), however, and since at least two other members of the grapevine ZIP gene family are also expressed during berry development (*VvZIP5.1* and *VvZIP6.1*; Figure 2), the putative role of VvZIP3 in Zn-uptake in these tissues need to be further analyzed.

### *The expression of VvZIP3 is induced by Zn in normal berries*

**Figure 8:**
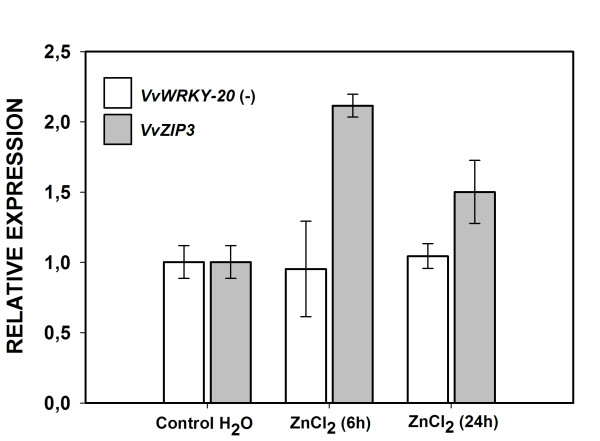
***VvZIP3*response under zinc treatment.** Expression analysis of *VvZIP3* in pre-veraison berries under Zn treatment (2 mM ZnCl_2_) at 6 and 24 hours. Expression in non-treated (H_2_O) pre-veraison berries samples for *VvZIP3* and *VvWRKY-20* (negative control), was adjusted to 1 relative unit. qPCR analyses of *VvZIP3 and VvWRKY-20* expression was normalized against the expression level of *VvGAPDH*. Data represent means ± SD (n = 3).

To test if the *VvZIP3* expression is Zn-dependant in reproductive tissues, normal berries at pre-veraison stage were exposed to Zn treatment. To reproduce *in planta* situation, exogenous Zn^2+^ was added by generating a capillary ion flux to the sink tissues through the berry peduncle (see Methods). After 6 hours of treatment, expression of *VvZIP3* was up-regulated about 2.1 fold compared to no-treated berries and this up-regulation was maintained until the end of the experiment (24 hours), while the negative control gene, *VvWRKY-20*, which encodes a putative zinc-finger transcription factor expressed in grapevine leaves (see Additional file 2), showed no alteration in its transcriptional level, along this treatment (Figure 8). This result suggests that the zinc flow to reproductive organs promotes its accumulation in these tissues by inducing *VvZIP3* expression.

## Discussion

Although several ZIP genes have been characterized in plants [15,23,37-40], to date, no ZIP gene has been isolated for *Vitis* species. The ZIP family of metal transporters shares several characteristics, including a molecular size between 36 and 39 kDa, 8 trans-membrane domains, a cytoplasmic ‘variable region’ localized between transmembrane domains 3 and 4 that provides a potential metal-binding domain and carboxy and amino termini located on the outer side of the targeted membrane [15,21,22]. Indeed, VvZIP3 displays all these structural characteristics allowing to be considered as a member of the ZIP family (Figure 3).

As shown in the phylogenetic tree (Figure 1), the predicted amino acid sequence of VvZIP3 was most closely related to AtZIP1, AtZIP3 and AtZIP5 (50–56% of identity) from *A. thaliana*[15,16,22] indicating that VvZIP3 and those proteins share, probably, a common evolutionary ancestor. All of these proteins are able to complement the growth of the yeast strain ZHY3. This yeast mutant type is very sensitive to Zn deprivation because of the mutation of their both high (ZRT1)- and low (ZRT2)-affinity Zn uptake systems [19,20]. Similar to the Arabidopsis ZIP proteins, the grape ZIP protein encoded by the cDNA of *VvZIP3* complemented the growth of this yeast mutant (Figure 5). Moreover, VvZIP3 was localized to the plasma membrane, as shown by the WOLF-pSORT II prediction and transient expression of a VvZIP3-mGFP5 fusion protein in onion epidermal cells (Figure 4A and 4B). Hence, these findings demonstrate that *VvZIP3* gene encodes a putative Zn transporter that may participate in the uptake of this element in *Vitis vinifera L.*.

The tissue-specific expression determined for grapevine ZIP encoding genes reflects the complexity of this gene family and suggests a differential gene regulation associated to the nutritional requirements of grapevine. In this regard, several *ZIP* genes identified in other species display diverse transcriptional profiles regarding tissue specificity and response to Zn status. For example, in rice, *OsZIP4* mRNA accumulates in the phloem cells of the stem as well as in the vascular bundles of the roots and leaves [2,32], *OsZIP1* mRNA accumulates in Zn-deficient roots and shoots while *OsZIP2* mRNA accumulates primarily in Zn-deficient roots [24]. In the model legume *Medicago truncatulaMtZIP1* transcripts were only detected in Zn-deficient roots and leaves [30], while *MtZIP2* gene was expressed in roots and stems, but not in leaves, and its transcriptional activity could be induced by Zn [31]. In addition, the *VvZIP3* counterparts identified in Arabidopsis (principally *AtZIP1AtZIP3* and *AtZIP5*) show a strong transcriptional activity in root tissue under Zn deficiency [15,22]. Compared to other *VvZIP* genes (i.e *VvZIP5.1*), *VvZIP3* is mainly transcribed in grapevine reproductive tissues under field conditions, more specifically in developing flowers and in the pericarp and/or skin of berries at early growth stages (Figure 2 and 6A). In these tissues, *VvZIP3* expression seems to be induced in response to Zn exposure as deduced from the experiments with normal berries at pre-veraison stage under exogenous Zn treatment (Figure 8).

In addition, and even when *VvZIP3* is not expressed in seeds, its transcriptional activity was consistently repressed in parthenocarpic non-seeded berries (Figure 6B). This down-regulation is not due to a non-specific disruptive effect on gene expression caused by seedlessness. When non-seeded/seeded expression ratio was determined for several transporter encoding genes both, up-regulated and down-regulated genes were identified (See Additional file 1). Interestingly, such analysis revealed that a gene coding for a putative grapevine boron transporter also appears strongly repressed in non-seeded berries. Like Zn, boron is also an essential micronutrient required for normal reproductive development in plants, and B deficiency has been associated to parthenocarpic berry development in grapevines [10,41]. Similar to *VvZIP3VvBOR1* is predominantly expressed in flowers at anthesis and normal berries at early growth stages, preceding a B accumulation in reproductive tissues [42]. This expression profile seems adequate to fulfill the B requirement for cross-linking of rhamnogalacturonane II, a polysaccharide essential for cell wall formation during pollen tube growth [43].

In a similar way, the *VvZIP3* expression profile, its straight correlation with the Zn accumulation pattern during development of reproductive organs (Figure 7), and it’s up-regulation in response to an increase in vascular Zn content (Figure 8), suggests a participation of VvZIP3 in the Zn loading during early reproductive developmental stages. It has been reported that alteration of the expression of ZIP transporters affects Zn distribution. Recently, it has been demonstrated that constitutive over-expression of the *OsZIP4* gene in transgenic rice plants confers disarrangement of Zn distribution in the transgenic plants [32]. In these regard, we can speculate that the alteration of *VvZIP3* expression during flowering and fertilization can modify the distribution, remobilization and availability of Zn, and hence affects normal reproductive development. Considering that Zn is essential for the stabilization of many proteins involved in development such as proteins containing DNA-binding Zn-finger motifs, RING fingers and LIM domains [1,7,10] and that Zn-finger transcription factors have been involved in the development and function of floral tissues such as anthers, tapetum, pollen and pistil secretory tissues in several plant species [1,7,44,45], it is plausible to propose that VvZIP3 may play a key role in both flower and normal fruit development.

## Conclusions

Considering that Zn deficiency produces several developmental problems in grapevines [10-12] and that no information is available regarding the specificity, regulation and function of any ZIP gene in *Vitis vinifera L.*, this work provides relevant information about the functional characterization of a putative Zn transporter identified in this species. Using a functional molecular approach, our results suggest that *VvZIP3* encodes a plasma membrane putative Zn transporter protein member of the ZIP gene family. *VvZIP3* is principally expressed in reproductive tissues, being strongly repressed in parthenocarpic seedless berries that present lower zinc accumulation, suggesting that it may participate in Zn uploading for normal berry development and that changes in its expression could affect zinc availability during this process.

## Methods

### Plant material

Grapevine (*Vitis vinifera* L. var. Carménère) grown under field conditions in a commercial vineyard in the Maule Valley (Central Chile) during three growing seasons (S1, S2 and S3) were used in this study. Zn nutritional status in plant leaves was monitored and corrected by foliar spray applications to maintain a Zn-sufficiency condition equivalent to a foliar concentration of 45–55 ppm. Random sampling of different organs was performed starting at early flowering until mature fruit stage (from October to April) from plants grown in the same plot. Stages to be sampled were defined according to the Modified Eichhorn-Lorenz System [46]. Flowering stages collected were: EL19, inflorescences or little clusters (LC) and EL 23, flowers at full bloom (F). Fruit developmental stages were: EL31, berries at preveraison 7 mm in diameter (PV); EL35, berries at veraison (V) and EL38, berries at postveraison harvest-ripe (M). For sampling, phenological stages were determined for normal seeded berries, then clusters were collected and seeded and non-seeded berries from the some bunch were separated for further processing.

### Identification and isolation of VvZIP3

From expression libraries made from RNA of normal and parthenocarpic *Vitis vinifera L.* cv. carménère berries (DEGECHIVID database; http://www.genomicafrutos.cl), an EST sequence highly similar to genes encoding Zn transporter proteins was obtained after comparison in the grapevine GENOSCOPE (http://www.genoscope.cns.fr/externe/GenomeBrowser/Vitis/) and NCBI (http://www.ncbi.nlm.nih.gov/genome/seq/BlastGen/BlastGen.cgi?taxid=29760) databases.

The sequence, named *VvZIP3*, was translated to obtain the open reading frame containing the initial methionine and the first stop codon using the OMIGA 2.0 software *Vitis vinifera L.*[47]. Identification of conserved domains in the predicted protein was carried out using InterProScan (Hunter, et al. 2009) and ScanProsite (de Castro, et al. 2006). Grand average hydropathy was obtained according to Kyte and Doolittle model [48]. Potential transmembrane domains in the predicted protein sequence were identified using TMPred [49], and TMHMM [50]. Signal peptides, as well as possible subcellular targeting sites were assessed using Wolf PSORT-II software [36]. Alignments were performed using ClustalW [51] and phylogenetic analyses were conducted with MEGA4 [52]. Phylogenetic trees were inferred using the Neighbor-Joining method [53]. The ZIP genes accession numbers used in tree construction are: *Vitis vinifera* (VvZIP1.1, GSVIVT00002087001; VvZIP1.2, GSVIVT00002088001; VvZIP2,GSVIVT00024285001; VvZIP3, GSVIVT00030117001; VvZIP4,GSVIVT00032208001; VvZIP5.1, GSVIVT00037538001; VvZIP5.2,GSVIVT00037540001; VvZIP6.1, GSVIVT00024060001; VvZIP6.2, GSVIVT00029326001; VvZIP7.1, GSVIVT00027686001; VvZIP7.2,GSVIVT00031911001; VvZIP8, GSVIVT00030650001; VvZIP9,GSVIVT00024638001; VvZIP10, GSVIVT00031915001; VvZIP11.1,GSVIVT00033348001; VvZIP11.2, GSVIVT00033353001; VvZIP11.3,GSVIVT00033352001; VvZIP11.4, GSVIVT00033350001; VvZIP12, GSVIVT00030116001; VvZIP13, GSVIVT00033649001) and *Arabidopsis thaliana* (AtZIP1, AT3G12750; AtZIP2, AT5G59520; AtZIP3, AT2G32270; AtZIP4, AT1G10970; AtZIP5, AT1G05300; AtZIP6, AT2G30080; AtZIP7, AT2G04032; AtZIP8, AT5G45105; AtZIP9, AT4G33020; AtZIP10, AT1G31260; AtZIP11, AT1G55910; AtZIP12, AT5G62160; AtZIP13, AT3G08650; AtIRT1, AT4G19690; AtIRT2, AT4G19680; AtIRT3, AT1G60960; AtIAR1, AT1G68100; AtGUFA, AT3G20870).

### Plant RNA extraction

Total RNA was extracted from 2 to 3 g of frozen (−80°C) roots, leaves, stems, little clusters, flowers, fruits and seeds (from pre-veraison to mature grapes) using the modified CTAB method [54]. Total RNA integrity was corroborated by formaldehyde agarose gel electrophoresis and their purity by OD_260/280_ ratio *>*1.95. Following DNase (DNAse I, Ambion) treatment of total RNA, first-strand cDNA synthesis was carried out from 2 μg of total RNA for each sample using oligo (dT) according to the manufacturer’s instructions (Revertaid First Strand cDNA Synthesis K1622 Kit, Fermentas).

### Gene expression analyses

Expression analysis was performed with three independent total RNA extractions (biological repeats). A standard curve was generated for each *ZIP* gene and *VvGAPDH* (as housekeeping gene) using a cDNA serial dilution. The resultant PCR efficiency calculations were imported into relative expression data analysis. PCR parameters used were: 94°C for 4 min; 94°C for 1 min, 60°C (annealing temperature) for 1 min, and 72°C for 1 min for 30 cycles; and a final step at 72°C for 7 min. The PCR products were visualized on agarose gels and isolated with the E.Z.N.A gel extraction kit (Omega Bio-Tek Inc.) to determine the primers efficiency. Gene transcript levels were measured by quantitative PCR (qPCR) using a DNA Engine Opticon 2 Cycler System (MJ Research). All reactions were performed using the Brilliant SYBR Green Master Mix (Stratagene) according to the procedure described by the manufacturer. For each sample, qPCR reactions were carried out in triplicate (technical repeats) using 10 μl Master Mix, 0.5 μl 250 nM each primer, 1 μl diluted cDNA and nuclease-free water in a final volume of 20 μl. Fluorescence was measured at the end of each amplification cycle. Amplification was followed by a melting curve analysis with continuous fluorescence data acquisition during the 65–95°C melt. The raw data were manually analyzed and expression was normalized to *GAPDH* gene (*VvGADPH*, NCBI/GenBank database accession number CN938023) *Ubiquitin* gene (*VvUBQ*, TIGR database accession number TC32075) to minimize variation in cDNA template levels. Primer sets used for qPCR were: VvZIP1.1rtFwd (5′-TGATATACATGGCGCTGG-3′) and VvZIP1.1rtRev (5′-CAGACACAAGAAAGAAAAGACG-3′) for *VvZIP1.1*; VvZIP2rtFwd (5′-CCATCAACCATCTCGTTGC-3′) and VvZIP2rtRev (5′-CAAACAAGGATCGTTTACAAGC-3′) for *VvZIP2*; VvZIP3rtFwd (5′-ACGACGAAAACAGCCCAAC-3′) and VvZIP3rtRev (5′-GGAGTCTCACATTGCTTTGC-3′) for *VvZIP3*; VvZIP4rtFwd (5′-CTGGTCATCGAAGGCATATTCG-3′) and VvZIP4rtRev (5′-AGGCCCCAAAACAAGAATTAGG-3′) for *VvZIP4*; VvZIP5.1rtFwd (5′-AAGTTGGAGAGCATGAAGG-3′) and VvZIP5.1rtRev (5′-ATTGGTGGAAAGTGAGAGC-3′) for *VvZIP5.1*; VvZIP6.1rtFwd (5′-GATGACAGTAGTGCGAATGC-3′) and VvZIP6.1rtRev (5′-TGGTCTCAACTCTCAACAAGC-3′) for *VvZIP6.1*; VvZIP8rtFwd (5′-AGGCCCTCTTCTTCAACTTCC-3′) and VvZIP8rtRev (5′-AGCCATGCCCAATATCAGC-3′) for *VvZIP8*; VvZIP11.1rtFwd (5′-CACCGGTATTGTCATAGATGC-3′) and VvZIP11.1rtRev (5′-GGAACACACTTCAAGATGAGC-3′) for *VvZIP11.1*; VvZIP13rtFwd (5′-GTCGACACATGGTCCTTCC-3′) and VvZIP13rtRev (5′-CCGCACTATTTTCCAAAAGC-3′) for *VvZIP13*; VvGAPDHFwd (5′-TTCCGTGTTCCTACTGTTG-3′) and VvGAPDHRev (5′-CCTCTGACTCCTCCTT GAT-3′) for *VvGAPDH*; VvUBQFwd (5′-GTGGTATTATTGAGCCATCCTT-3′) and VvUBQRev (5′-AACCTCCAATCCAGTCATCTAC-3′) for *VvUBQ*. For the Zn treatment assay in pre-veraison berries, a negative control gene, VvWRKY-20 (WRKY transcription factor 20-like, GSVIVT01030046001), whose expression is not altered during metal treatments (unpublished data) was included. The primers for VvWRKY-20 were: VvWRKY-20Fwd (5′-CAACAAACTCCAAGTGCAGAACC-3′) and VvWRKY-20Rev (5′-CACCCCCAAAAAATGAGAAGG-3′).

### Zinc Treatment

In order to determine whether *VvZIP3* expression is affected by Zn, *Vitis vinifera* L. cv. Carménère grape clusters were harvested from field-grown vines in a commercial vineyard in the Maule Valley (Central Chile) during the season S3 at the preveraison stage (EL31, PV) [46]. Uniform berries with their respective peduncles were excised under water, and were positioned on perforated plastic trays (Kim trak 25 × 14 cm) so that the cut pedicels protruded through the holes into a dish containing the proper solution [55]. The experimental conditions were: temperature at 25°C and light at 156 W cm-2. 2 mM of ZnCl_2_ was applied as a dip solution throughout the experiment and distilled water as a control. Eight random berries from each tray were collected at 6 and 24 hours, pooled together and processed for RNA extraction. The experiment included three trays per treatment (Zn and water) and was repeated twice.

### Determination of Zn content

Total Zn content was determined in little clusters, flowers and fruits at different developmental stages (pre-veraison, veraison and mature grapes) according to Karla [56]. The reagents used were of high purity (Suprapur, Merck, Darmstadt). The cleaning of the material was fundamental to guarantee the optimum result in analysis. Tissues were washed with deionized water and oven-dried (80°C) to constant weight. The samples were subsequently homogenized and kept in plastic containers for later analysis. Dried tissues were ground into powder, then ashed at 500°C and dissolved in 2 M HCl [56]. The resulting solution was filtered and washed with bidistilled water to a final volume of 50 mL in a pre-treated volumetric flask. The analyses were done in duplicate. The measurements were done by flame atomic absorption spectroscopy (air/acetylene), using a Unicam 969 spectrophotometer with deuterium background corrector. The method of analysis was validated using the SRM-1570 certified reference material (spinach), supplied by the National Institute of Standards and Technology (USA). Replications of the reference material showed good exactness with relative errors varying between 2.2 – 3.4%.

### Yeast complementation assay

The following strains of the yeast *Saccharomyces cerevisiae* were used in this study: wild type parent strain DY1457 (*MAT*a, *ade6, can1, ura3, leu2, his3, trp1*) and the Zn^2+^ uptake defective double mutant ZHY3 Δzrt1/Δzrt2 (MATa*, his3, leu2, met1, lys2, ura3, zrt1, zrt2*) [19,20]. Growth occurred in yeast potato dextrose or in synthetic defined (SD) medium with 2% (w/v) glucose or galactose, supplemented as necessary [57]. For metal complementation assay, yeast was grown in liquid SD medium (with 2% [w/v] galactose) until OD = 1, and drop assays were performed on SD plates containing different concentrations of Zn (10 μM, 100 μM, 750 μM and 2 mM). Yeast cells were transformed using the S.c. EasyComp Transformation Kit (Invitrogen). The *VvZIP3* cDNA was subcloned from the TOPO vector clone (TOPO TA Cloning Vector System, Invitrogen) into the pYES2 plasmid (Invitrogen) using the primers VvZIP3-fwd (5-CATCTGGATCCATGAGCAAGCTTCAGTTCTATCCAT-3) and VvZIP3-rev (5-CCATCTCTCGAGGATTCCACCCCATTTGGCCAGA-3) to introduce the *BamH*I and *Xho*I sites (underlined).

### Transient expression assay in onion epidermal cells

The intracellular localization of VvZIP3 was determined by monitoring the transient expression of a VvZIP3-mGFP5 translational fusion product in onion epidermal cells after DNA particle bombardment. The coding region of mGFP5, a green fluorescent protein modified for plants [58] was fused to the *Xba*I site of the pART7 vector [59]. The *VvZIP3* cDNA was subcloned from pYES2-VvZIP3 to the *Xho*I-*Kpn*I–digested pART7 between the 35 S cauliflower mosaic virus promoter and the octopine synthase terminator and in frame with *mGFP5*, using the VvZIP3XhoI-fwd (5-CCATCTCTCGAGATGAGCAAGCTTCAGTTCTATC-3) and VvZIP3KpnI-rev (5-CCCCGGTACCCGATTCCACCCCATTTGGCCAGA-3) primers (sites underlined). Gold particles (0.6 μm) coated with the resulting construct was delivered into onion bulb scale epidermal cell layers with a PDS-1000/He Particle Delivery System (Bio-Rad). The bombardment parameters were as follows: discharge pressure of 1100 p.s.i. with a 900 p.s.i. rupture disk, and distance to target tissue of 6 cm. Onion epidermal layers were placed onto MS agar plates before bombardment and incubated at 22°C for 24 h after particle delivery. Viewing of bombarded samples was done with a confocal microscope (Zeiss LSM-510 system).

## Authors’ contributions

FGC participated in the design of the study, carried out the experiments, interpreted the data, and drafted the manuscript. RP carried out part of the qPCR experiment. RPC carried out part of the sequence analyses and gave extensive advices on qPCR data analysis. JT carried out the metal determination experiments. HPC directed the overall project and helped to draft the manuscript. JC participated in the design of the study and helped to draft the manuscript. SG carried out Zn treatment and gene expression analysis. SRL and EG conceived the research, participated in the design of the study and played a major part in results interpretation. All authors read and approved the final manuscript.

## Supplementary Material

Additional file 1 **NS/S Expression ratio of different ESTs associated to transport.** Putative genes associated to transport that showing a significant expression variation (p-value < 0.05) from expression libraries from parthenocarpic (NS) and normal (S) *Vitis vinifera L.* cv. carménère berries (DEGECHIVID database; http://www.genomicafrutos.cl). Click here for file

Additional file 2 **A)** Comparison of the VvWRKY-20 amino acid sequence with its putative homologue AtZAP1. Alignment was performed with the highest homologous sequence from Vitis vinifera L. Genome to AtZAP1 (AT2G04880), named VvWRKY-20 (GSVIVT01030046001, http://www.genoscope.cns.fr). Alignments were performed using ClustalW. The WRKY motifs are highlight in yellow boxes and a blue underline indicate the DNA-binding motifs. Identical residues are blackened; similar residues are highlight in grey. **B)** Gene expression analysis of VvWRKY-20 in grapevine tissues. Expression profiles of VvWRKY-20 in R (roots); L (leafs); S (stems); LC (little clusters); F (flowers); PV (pre-veraison fruit); V (whole veraison fruit); M (whole mature fruit). Expression in root samples was adjusted to 1 relative unit. Data represent means ± SD (n = 3). Click here for file
