# Supplementary material for: Characterization of a putative grapevine Zn transporter, VvZIP3, suggests its involvement in early reproductive development in Vitis vinifera L
Source: BMC Plant Biol. 2012 Jul 23;12:111. doi: 10.1186/1471-2229-12-111 (PMC3432002; doi:10.1186/1471-2229-12-111)
Supplement: Additional file 1 — NS/S Expression ratio of different ESTs associated to transport. Putative genes associated to transport that showing a significant expression variation (p-value < 0.05) from expression libraries from parthenocarpic (NS) and normal (S) Vitis vinifera L. cv. carménère berries (DEGECHIVID database; http://www.genomicafrutos.cl). [file 1471-2229-12-111-S1.pdf]

Additional file 1:

NS/S Expression ratio of different ESTs associated to transport.

EST code	Function	p-value	NS/S Expression ratio
VVCCGC1046C09.g	**Homologue to aquaporin [Vitis vinifera]**	0.0002	**8.79**
VVCCGS1050B10.g	**Similar to putative Na+/H+-exchanging protein [Arabidopsis thaliana]**	0.0163	**7.38**
VVCCGS1066B01.g	**Homologue to ABC-type Fe3+ transport system [Pseudomonas fluorescens PfO-1]**	0.0006	**6.12**
VVCCGC2021A03.b	**Similar to putative amino acid carrier [Oryza sativa (japonica cultivar-group)]**	0.0195	**4.82**
VVCCGC1054H10.g	**Homologue to putative aquaporin PIP1-1 [Vitis berlandieri x Vitis rupestris]**	0.0005	**3.63**
VVCCGC3021C01.b	**Homologue to putative aquaporin [Vitis vinifera]**	0.0063	**3.35**
VVCCGC3048C02.b	**Homologue to putative aquaporin PIP2-2 [Vitis berlandieri x Vitis rupestris]**	0.0104	**2.95**
VVCCGS1063D10.g	**Similar to sulfate transporter [Arabidopsis thaliana], partial**	0.0052	**2.69**
VVCCGC2069C05.b1	**Similar to outward-rectifying potassium channel KCO1 [Eucalyptus camaldulensis]**	0.0262	**2.48**
VVCCGC3050D04.b	**Similar to ammonium transporter (AMT1.1) [Lotus corniculatus var. japonicus]**	0.0324	**2.20**
VVCCGS1044E03.g	**Homologue to putative sucrose transporter [Vitis vinifera]**	0.0054	**1.69**
VVCCGS1061E01.g	**Similar to metal transporter, putative (ZIP4) [Arabidopsis thaliana]**	0.0206	**1.54**
VVCCGC1059G04.g	**Similar to aquaporin [Vitis vinifera]**	0.0056	**1.42**
VVCCGS2246F08.b	**Similar to putative copper-transporting P-type ATPase [Oryza sativa (japonica cultivar-group)]**	0.0096	**0.56**
VVCCGC1009E07.g	**Homologue to putative aquaporin PIP1-3 [Vitis berlandieri x Vitis rupestris]**	0.0376	**0.51**
VVCCGC1029A05.g	**Similar to sulfate transporter-like protein [Oryza sativa (japonica cultivar-group)]**	0.0046	**0.48**
VVCCGS2225E04.b	**Similar to putative aquaporin TIP3 [Vitis berlandieri x Vitis rupestris]**	0.0026	**0.36**
VVCCGS1028B01.g	**Homologue to aquaporin [Vitis vinifera]**	0.0142	**0.24**
VVCCGS2204F01.b	**Homologue to aquaporin [Vitis vinifera]**	0.0239	**0.23**
VVCCGC1012H02.g	**Similar to putative permease 1 [Arabidopsis thaliana]**	0.0099	**0.23**
VVCCGC1071D08.g	**Similar to metal transport protein [Medicago truncatula]**	0.0079	**0.19**
VVCCGS1028A01.g	**Similar to phosphate transporter [Lycopersicon esculentum]**	<.0001	**0.18**
VVCCGS1027H10.g	**Similar to ABC transporter [Gossypium hirsutum]**	0.0131	**0.18**
VVCCGS1009B03.g	**Similar to boron transporter [Oryza sativa (japonica cultivar-group)]**	0.0208	**0.16**
VVCCGS1006G05.g	**Similar to metal transport protein [Medicago truncatula]**	0.0049	**0.14**
VVCCGS2230B05.b	**Similar to putative anthocyanin permease [Lycopersicon esculentum]**	0.0057	**0.04**

Putative genes associated to transport that showing a significant expression variation (p-value < 0.05) from expression libraries from parthenocarpic (NS) and normal (S) *Vitis vinifera* cv. carménère berries (DEGECHIVID database; www.genomicafrutos.cl).
