# Supplementary material for: Characterization of a putative grapevine Zn transporter, VvZIP3, suggests its involvement in early reproductive development in Vitis vinifera L
Source: BMC Plant Biol. 2012 Jul 23;12:111. doi: 10.1186/1471-2229-12-111 (PMC3432002; doi:10.1186/1471-2229-12-111)
Supplement: Additional file 2 — A) Comparison of the VvWRKY-20 amino acid sequence with its putative homologue AtZAP1. Alignment was performed with the highest homologous sequence from Vitis vinifera L. Genome to AtZAP1 (AT2G04880), named VvWRKY-20 (GSVIVT01030046001, http://www.genoscope.cns.fr). Alignments were performed using ClustalW. The WRKY motifs are highlight in yellow boxes and a blue underline indicate the DNA-binding motifs. Identical residues are blackened; similar residues are highlight in grey. B) Gene expression analysis of VvWRKY-20 in grapevine tissues. Expression profiles of VvWRKY-20 in R (roots); L (leafs); S (stems); LC (little clusters); F (flowers); PV (pre-veraison fruit); V (whole veraison fruit); M (whole mature fruit). Expression in root samples was adjusted to 1 relative unit. Data represent means ± SD (n = 3). [file 1471-2229-12-111-S2.pdf]

Additional File 2:

A) Comparison of the VvWRKY-20 amino acid sequence with its putative ortholog AtZAP1.

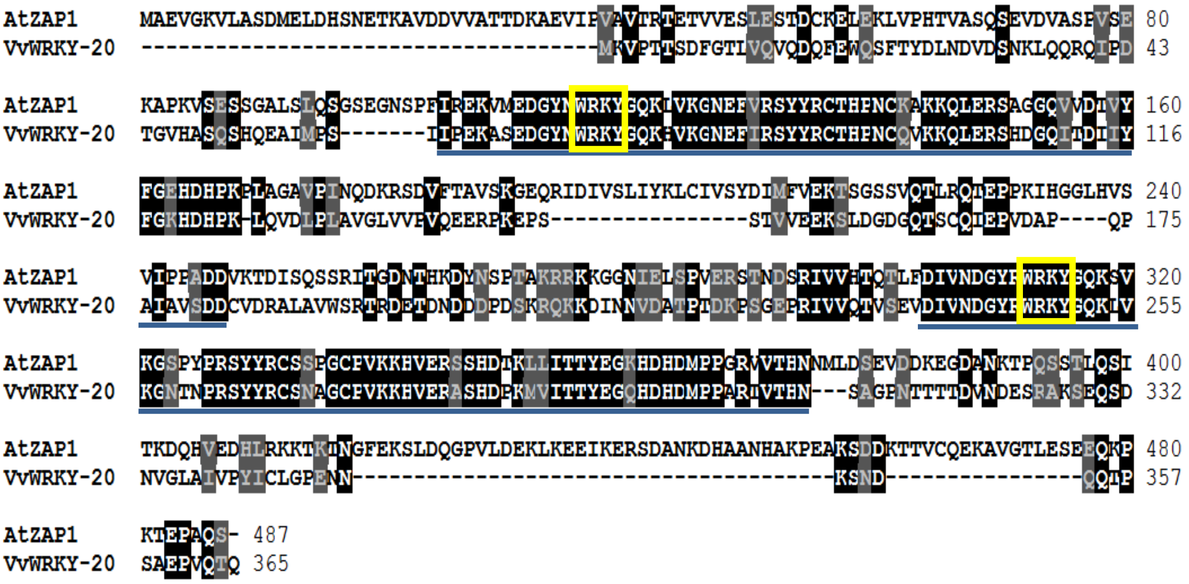

Alignment was performed with the highest homologous sequence from Vitis vinifera L. Genome to AtZAP1 (AT2G04880), named VvWRKY-20 (GSVIVT01030046001, http://www.genoscope.cns.fr). Alignments were performed using ClustalW. The WRKY motifs are highlighted in yellow boxes and a blue underline indicates the DNA-binding motifs. Identical residues are blackened and similar residues are highlighted in grey.

B) Gene expression analysis of *VvWRKY-20* in grapevine tissues.

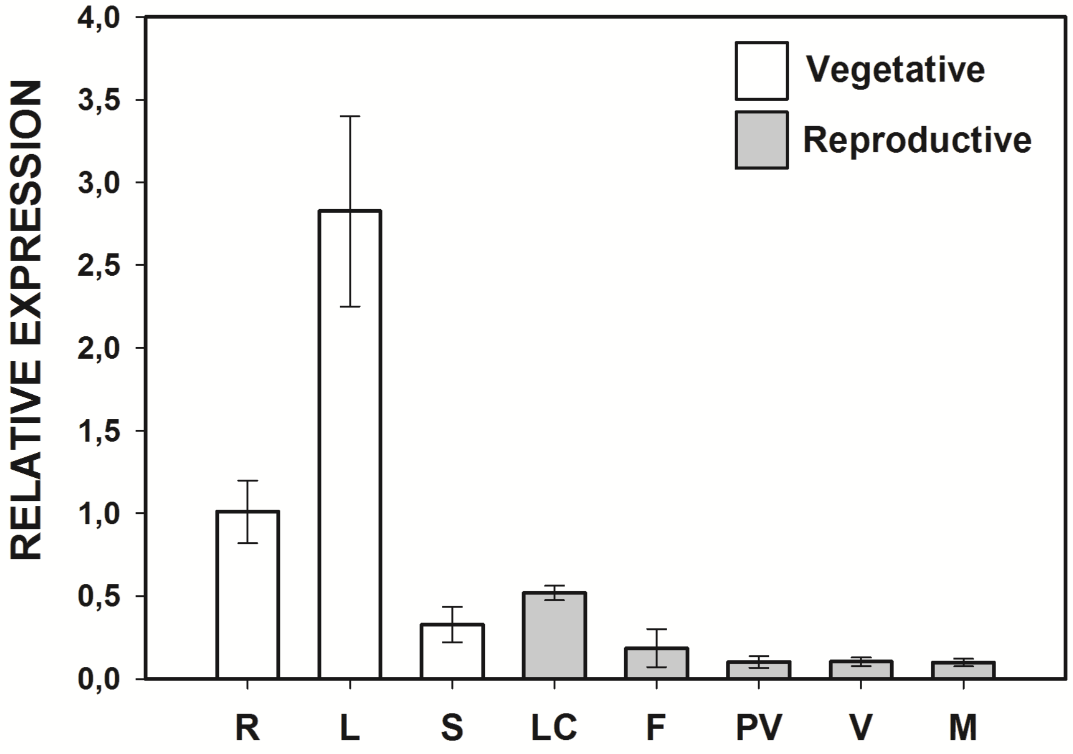

Expression profiles of *VvWRKY-20* in R (roots); L (leafs); S (stems); LC (little clusters); F (flowers); PV (pre-veraison fruit); V (whole veraison fruit); M (whole mature fruit). Expression in root samples was adjusted to 1 relative unit. Data represent means ± SD (n = 3).
